# Supplementary material for: The transdisciplinary research process and participatory research approaches used in the field of neglected tropical diseases: A scoping review
Source: PLoS Negl Trop Dis. 2025 Apr 1;19(4):e0012959. doi: 10.1371/journal.pntd.0012959 (PMC11977956; doi:10.1371/journal.pntd.0012959)
Supplement: S1 Appendix — (DOCX) [file pntd.0012959.s001.docx]

**S1 Appendix**

**Working definitions of standard terms used in the study**

| Terms | Definitions |
| --- | --- |
| Transdisciplinary Research Process (TDR) | ‘Acknowledging the absence of a universal definition for TDR, this study considered an article as TDR if it had a shared goal to solve complex real-world problems through the integration of diverse knowledge systems and collaboration between scientific, societal, and non-academic stakeholders and must specifically include individuals or their communities directly impacted by NTDs’ (1–4). |
| Participatory Research | ‘Participatory research is a research-to-action approach that prioritises the **active involvement and empowerment of affected individuals and communities** to ensure that solutions are contextually relevant and equitable, rooted in their own experiences and needs. Local knowledge and perspectives are not only acknowledged but form the basis for research and planning’ (5). |
| One Health | ‘One Health is a collaborative, multisectoral, and transdisciplinary approach—working at the local, regional, national, and global levels—with the goal of achieving optimal health outcomes recognising the interconnection between people, animals, plants, and their shared environment’ (6). |
| Scientific actors (academic actors) | ‘Scientific or academic researchers from unrelated disciplines’ (7)  ‘Core scientists are the main scientific actors throughout the course of a project, and the scientific consultants support the core group; they are academic experts, often from external organisations, who offer expertise that is not well enough covered by the core team’ (8). |
| Societal actors and non-academic actors | ‘Societal and non-academic actors fall into one of four categories: private sector (i.e., business and industry), public sector (*i.e.*, government and civil service), civic sector (i.e., civil society and non-governmental organisations), and citizens/communities themselves. Traditional and modern media may also play an important role. Many non-academic actors have no formal science education, but they are experts in their  respective fields and can provide insights that go beyond academic knowledge’ (7). |
| Neglected Tropical Diseases (NTDs) | ‘NTDs are a diverse group of 20 diseases and conditions that are mainly prevalent in tropical areas, where they mostly affect marginalised communities, causing devastating health, social and economic consequences to more than one billion people. The epidemiology of NTDs is complex, often related to environmental conditions and associated with complex life cycles. All these factors make their public health control challenging’ (9). |
| Complex problems | ‘Complex problems are unique, and usually long term and the solutions to solve a problem cannot be applied to other like problems. They require learning with many factors interacting not only with the subject but also with each other.’ (10) |
| Power Dynamics | ‘Power dynamics refer to the inherent structures and influences of power that exist between individuals and groups within a given context. This idea extends beyond mere authority or control, delving into more nuanced territories of influence, dominance, privilege, and communication styles. In its simplest form, power can be understood as the ability to influence or control outcomes.’ (11) |

References:

1. Klein JT, Grossenbacher-Mansuy W, Häberli R, Bill A, Scholz R, Welti M. Transdisciplinarity: Joint Problem Solving among Science, Technology, and Society An Effective Way for Managing Complexity. 2001.

2. Lang DJ, Wiek A, Bergmann M, Stauffacher M, Martens P, Moll P, et al. Transdisciplinary research in sustainability science: practice, principles, and challenges. Sustain Sci. 2012 Feb 1;7(1):25–43.

3. Jahn T, Bergmann M, Keil F. Transdisciplinarity: Between mainstreaming and marginalization. Ecological Economics. 2012 Jul 1;79:1–10.

4. Pohl C. From science to policy through transdisciplinary research. Environmental Science & Policy. 2008 Feb;11(1):46–53.

5. Cornwall A, Jewkes R. What is participatory research? Social Science & Medicine. 1995 Dec 1;41(12):1667–76.

6. Mackenzie JS, Jeggo M. The One Health Approach—Why Is It So Important? Trop Med Infect Dis. 2019 May 31;4(2):88.

7. OECD. Addressing societal challenges using transdisciplinary research [Internet]. 2020 Jun [cited 2021 Jul 12]. (OECD Science, Technology and Industry Policy Papers; vol. 88). Report No.: 88. Available from: https://www.oecd-ilibrary.org/science-and-technology/addressing-societal-challenges-using-transdisciplinary-research_0ca0ca45-en

8. Enengel B, Muhar A, Penker M, Freyer B, Drlik S, Ritter F. Co-production of knowledge in transdisciplinary doctoral theses on landscape development—An analysis of actor roles and knowledge types in different research phases. Landscape and Urban Planning. 2012 Mar;105(1–2):106–17.

9. WHO. Neglected tropical diseases -- Global [Internet]. WHO; [cited 2022 Jan 30]. Available from: https://www.who.int/health-topics/neglected-tropical-diseases#tab=tab_1

10. Glouberman S, Zimmerman B. Complicated and Complex Systems: What Would Successful Reform of Medicare Look Like? Commission on the Future of Health Care in Canada: Discussion Paper No 8. 2002 Jan 1;8.

11. ATLAS.ti [Internet]. [cited 2024 Jul 1]. Power Dynamics in Research| Definition, Examples & Awareness. Available from: https://atlasti.com/guides/qualitative-research-guide-part-1/power-dynamics
