# Supplementary material for: The transdisciplinary research process and participatory research approaches used in the field of neglected tropical diseases: A scoping review
Source: PLoS Negl Trop Dis. 2025 Apr 1;19(4):e0012959. doi: 10.1371/journal.pntd.0012959 (PMC11977956; doi:10.1371/journal.pntd.0012959)
Supplement: S2 Appendix — (DOCX) [file pntd.0012959.s002.docx]

**S2 Appendix**

Search strategy per database

## Table 1: Search strategy in PubMed

| **Search** | **Query** | **Results** |
| --- | --- | --- |
| **#3** | **#1 AND #2** | **821** |
| **#2** | **"Trans Disciplinary Research*"[tiab] OR "Transdisciplinary Research*"[tiab] OR "transdisciplinarit*"[tiab] OR "social research*"[tiab] OR "participation research*"[tiab] OR "action research*"[tiab] OR "one health*"[tiab] OR "integrated learning action research"[tiab] OR "stakeholder*"[tiab] OR "mutual learning"[tiab] OR "co creation*"[tiab] OR "cocreation*"[tiab]** | **64,373** |
| **#1** | **"Neglected Diseases"[Mesh] OR "neglected tropical dis*"[tiab] OR "SCHISTOSOMIASIS"[tiab] OR "HELMINTHIASIS"[tiab] OR "Leprosy"[Mesh] OR "Leprosy"[tiab] OR "Hansen s Dis*"[tiab] OR "Hansens Dis*"[tiab] OR "Hansen Dis*"[tiab] OR "LYMPHATIC FILARIASIS"[tiab] OR "ONCHOCERCIASIS"[tiab] OR "TRACHOMA"[tiab] OR "plague*"[tiab] OR "Buruli Ulcer*"[tiab] OR "Chagas Dis*"[tiab] OR "Cysticercosis"[tiab] OR "Dengue Fever"[tiab] OR "Dracunculiasis"[tiab] OR "Guinea Worm Dis*"[tiab] OR "Echinococcosis"[tiab] OR "Fascioliasis"[tiab] OR "Human African Trypanosomiasis"[tiab] OR "African Sleeping Sickness"[tiab] OR "Leishmaniasis"[tiab] OR "Lymphatic Filariasis"[tiab] OR "Mycetoma"[tiab] OR "Onchocerciasis"[tiab] OR "Rabies"[tiab] OR "Ascaris"[tiab] OR "Hookworm*"[tiab] OR "Whipworm*"[tiab] OR "Trachoma*"[tiab]** | **174,887** |

## Table 2: Search strategy in Embase.com

| **Search** | **Query** | **Results** |
| --- | --- | --- |
| **#3** | #1 AND #2 | **1,079** |
| **#2** | (‘Trans Disciplinary Research*’ OR ‘Transdisciplinary Research*’ OR ‘transdisciplinarit*’ OR ‘social research*’ OR ‘participation research*’ OR ‘action research*’ OR ‘one health*’ OR ‘integrated learning action research’ OR ‘stakeholder*’ OR ‘mutual learning’ OR ‘co creation*’ OR ‘cocreation*’):ti,ab,kw | **82,012** |
| **#1** | 'neglected disease'/exp OR 'African trypanosomiasis'/exp OR 'Buruli ulcer'/exp OR 'leprosy'/exp OR 'cholera'/exp OR 'lymphatic filariasis'/exp OR 'onchocerciasis'/exp OR (‘cholera’ OR ‘neglected tropical dis*’ OR ‘SCHISTOSOMIASIS’ OR ‘HELMINTHIASIS’ OR ‘Leprosy’ OR ‘Hansen s Dis*’ OR ‘Hansens Dis*’ OR ‘Hansen Dis*’ OR ‘LYMPHATIC FILARIASIS’ OR ‘ONCHOCERCIASIS’ OR ‘TRACHOMA’ OR ‘plague*’ OR ‘Buruli Ulcer*’ OR ‘Chagas Dis*’ OR ‘Cysticercosis’ OR ‘Dengue Fever’ OR ‘Dracunculiasis’ OR ‘Guinea Worm Dis*’ OR ‘Echinococcosis’ OR ‘Fascioliasis’ OR ‘Trypanosomiasis’ OR ‘African Sleeping Sickness’ OR ‘Leishmaniasis’ OR ‘Lymphatic Filariasis’ OR ‘Mycetoma’ OR ‘Onchocerciasis’ OR ‘Rabies’ OR ‘Ascaris’ OR ‘Hookworm*’ OR ‘Whipworm*’ OR ‘Trachoma*’):ti,ab,kw | **238,754** |

Table 3: Search strategy in Clarivate Analytics/Web of Science Core Collection

| **Search** | **Query** | **Results** |
| --- | --- | --- |
| **#3** | #1 AND #2 | **1,064** |
| **#2** | TS=(“Trans Disciplinary Research*” OR “Transdisciplinary Research*” OR “transdisciplinarit*” OR “social research*” OR “participation research*” OR “action research*” OR “one health*” OR “integrated learning action research” OR “stakeholder*” OR “mutual learning” OR “co creation*” OR “cocreation*”) | **159,948** |
| **#1** | TS=(“cholera” OR “neglected tropical dis*” OR “SCHISTOSOMIASIS” OR “HELMINTHIASIS” OR "Leprosy" OR “Hansen s Dis*” OR “Hansens Dis*” OR “Hansen Dis*” OR “LYMPHATIC FILARIASIS” OR “ONCHOCERCIASIS” OR “TRACHOMA” OR “plague*” OR “Buruli Ulcer*” OR “Chagas Dis*” OR “Cysticercosis” OR “Dengue Fever” OR “Dracunculiasis” OR “Guinea Worm Dis*” OR “Echinococcosis” OR “Fascioliasis” OR “Human African Trypanosomiasis” OR “African Sleeping Sickness” OR “Leishmaniasis” OR “Lymphatic Filariasis” OR “Mycetoma” OR “Onchocerciasis” OR “Rabies” OR “Ascaris” OR “Hookworm*” OR “Whipworm*” OR “Trachoma*”) | **204,136** |

Table 4: Search strategy in Cumulative Index to Nursing and Allied Health Literature (CINAHL)

| **Search** | **Query** | **Results** |
| --- | --- | --- |
| **#3** | #1 AND #2 | **107** |
| **#2** | TI(“Trans Disciplinary Research*” OR “Transdisciplinary Research*” OR “transdisciplinarit*” OR “social research*” OR “participation research*” OR “action research*” OR “one health*” OR “integrated learning action research” OR “stakeholder*” OR “mutual learning” OR “co creation*” OR “cocreation*”) OR AB(“Trans Disciplinary Research*” OR “Transdisciplinary Research*” OR “transdisciplinarit*” OR “social research*” OR “participation research*” OR “action research*” OR “one health*” OR “integrated learning action research” OR “stakeholder*” OR “mutual learning” OR “co creation*” OR “cocreation*”) OR KW(“Trans Disciplinary Research*” OR “Transdisciplinary Research*” OR “transdisciplinarit*” OR “social research*” OR “participation research*” OR “action research*” OR “one health*” OR “integrated learning action research” OR “stakeholder*” OR “mutual learning” OR “co creation*” OR “cocreation*”) | **32,949** |
| **#1** | (MH "Neglected Diseases") OR TI(“cholera” OR “neglected tropical dis*” OR “SCHISTOSOMIASIS” OR “HELMINTHIASIS” OR "Leprosy" OR “Hansen s Dis*” OR “Hansens Dis*” OR “Hansen Dis*” OR “LYMPHATIC FILARIASIS” OR “ONCHOCERCIASIS” OR “TRACHOMA” OR “plague*” OR “Buruli Ulcer*” OR “Chagas Dis*” OR “Cysticercosis” OR “Dengue Fever” OR “Dracunculiasis” OR “Guinea Worm Dis*” OR “Echinococcosis” OR “Fascioliasis” OR “Human African Trypanosomiasis” OR “African Sleeping Sickness” OR “Leishmaniasis” OR “Lymphatic Filariasis” OR “Mycetoma” OR “Onchocerciasis” OR “Rabies” OR “Ascaris” OR “Hookworm*” OR “Whipworm*” OR “Trachoma*”) OR AB(“cholera” OR “neglected tropical dis*” OR “SCHISTOSOMIASIS” OR “HELMINTHIASIS” OR "Leprosy"[Mesh] OR "Leprosy" OR “Hansen s Dis*” OR “Hansens Dis*” OR “Hansen Dis*” OR “LYMPHATIC FILARIASIS” OR “ONCHOCERCIASIS” OR “TRACHOMA” OR “plague*” OR “Buruli Ulcer*” OR “Chagas Dis*” OR “Cysticercosis” OR “Dengue Fever” OR “Dracunculiasis” OR “Guinea Worm Dis*” OR “Echinococcosis” OR “Fascioliasis” OR “Human African Trypanosomiasis” OR “African Sleeping Sickness” OR “Leishmaniasis” OR “Lymphatic Filariasis” OR “Mycetoma” OR “Onchocerciasis” OR “Rabies” OR “Ascaris” OR “Hookworm*” OR “Whipworm*” OR “Trachoma*”) OR KW(“cholera” OR “neglected tropical dis*” OR “SCHISTOSOMIASIS” OR “HELMINTHIASIS” OR "Leprosy"[Mesh] OR "Leprosy" OR “Hansen s Dis*” OR “Hansens Dis*” OR “Hansen Dis*” OR “LYMPHATIC FILARIASIS” OR “ONCHOCERCIASIS” OR “TRACHOMA” OR “plague*” OR “Buruli Ulcer*” OR “Chagas Dis*” OR “Cysticercosis” OR “Dengue Fever” OR “Dracunculiasis” OR “Guinea Worm Dis*” OR “Echinococcosis” OR “Fascioliasis” OR “Human African Trypanosomiasis” OR “African Sleeping Sickness” OR “Leishmaniasis” OR “Lymphatic Filariasis” OR “Mycetoma” OR “Onchocerciasis” OR “Rabies” OR “Ascaris” OR “Hookworm*” OR “Whipworm*” OR “Trachoma*”) | **16,386** |
