# Supplementary material for: The transdisciplinary research process and participatory research approaches used in the field of neglected tropical diseases: A scoping review
Source: PLoS Negl Trop Dis. 2025 Apr 1;19(4):e0012959. doi: 10.1371/journal.pntd.0012959 (PMC11977956; doi:10.1371/journal.pntd.0012959)
Supplement: S3 Appendix — (DOCX) [file pntd.0012959.s003.docx]

**S3** **Appendix**

**Included studies in the review**

This appendix lists the studies included in the review, providing full citations for each source.

1. Ackumey MM, Kwakye-Maclean C, Ampadu EO, de Savigny D, Weiss MG. Health services for Buruli ulcer control: lessons from a field study in Ghana. PLOS NTD 2011;5(6). e1187, <https://doi.org/10.1371/journal.pntd.0001187>
2. Apte H, Chitale M, Das S, Manglani PR, Mieras LF. Acceptability of contact screening and

single dose rifampicin as chemoprophylaxis for leprosy in Dadra and Nagar Haveli, India. Leprosy Review. 2019;90(1):31–45. DOI: [10.47276/lr.90.1.31](https://doi.org/10.47276/lr.90.1.31)

1. Awah PK, Boock AU, Mou F, Koin JT, Anye EM, Noumen D, et al. Developing a Buruli ulcer community of practice in Bankim, Cameroon: A model for Buruli ulcer outreach in Africa. PLOS Neglected Tropical Diseases 2018;12(3). https://doi.org/10.1371/journal.pntd.0006238
2. Beran D, Lazo-Porras M, Cardenas MK, Chappuis F, Damasceno A, Jha N, et al. Moving from formative research to co-creation of interventions: insights from a community health system project in Mozambique, Nepal and Peru. BMJ Glob Health 2018;3(6):e001183.

doi:10.1136/bmjgh-2018-001183

1. Degeling C, Brookes V, Lea T, Ward M. Rabies response, One Health and more-than-human considerations in Indigenous communities in northern Australia. Research Online: [https://ro.uow.edu.au/sspapers/4089 2018; 212:](https://ro.uow.edu.au/sspapers/4089%202018;%20212) 60-67.
2. El Katsha S, Watts S, Khairy A, El-Sebaie O. Community participation for schistosomiasis control: a participatory research project in egypt. Int Q Community Health Educ. 1993;14(3):245-56. doi: 10.2190/9CBL-DWAP-63TB-H4JD.
3. Freudenthal S, Ahlberg BM, Mtweve S, Nyindo P, Poggensee G, Krantz I. School-based prevention of schistosomiasis: initiating a participatory action research project in northern Tanzania. Acta Trop. 2006;100(1-2):79-87. doi:10.1016/j.actatropica.2006.09.013. Epub 2006 13 Nov.
4. Gautam V, Bhardwaj P, Saxena D, Kumar N, S D. Multisectoral approach to achieve canine rabies controlled zone using Intervention Mapping: Preliminary results. PLOS ONE 2020;15(12) https://doi.org/10.1371/journal.pone.0242937
5. Jaeggi T, Manickam P, Weiss MG, Gupte MD. Stakeholders perspectives on perceived needs and priorities for leprosy control and care, Tamil Nadu, India. Indian J Lepr 2012, 84 : 177-184, PMID: 23484332
6. Kuipers P, Joy A, John A, Raju MS. A pilot study using participatory, translational, social science research methods to explore stakeholder perspectives on preventing delayed diagnosis in leprosy. Lepr Rev 2018; 89 (2), 124– 138, DOI: [10.47276/lr.89.2.124](https://doi.org/10.47276/lr.89.2.124)
7. Means AR, Jacobson J, Mosher AW, Walson JL. Integrated Healthcare Delivery: A Qualitative Research Approach to Identifying and Harmonizing Perspectives of Integrated Neglected Tropical Disease Programs. PLoS Negl Trop Dis 2016 ; 10(10): e0005085. <https://doi.org/10.1371/journal.pntd.0005085>
8. Onasanya A, Keshinro M, Oladepo O, Van Engelen J, Diehl JC. A Stakeholder Analysis of Schistosomiasis Diagnostic Landscape in South-West Nigeria: Insights for Diagnostics Cocreation. Front. Public Health, 2020; 8, [https://doi.org/10.3389/fpubh.2020.564381.](https://doi.org/10.3389/fpubh.2020.564381)
9. Ozano K, Dean L, Adekeye O, Bettee AK, Dixon R, Gideon NU, et al. Guiding principles for quality, ethical standards and ongoing learning in implementation research: multicountry learnings from participatory action research to strengthen health systems. Health Policy and Planning, 2020; 35 (supplement_2), ii137–ii149 doi: 10.1093/heapol/czaa123
10. Peters R, Lusli M, Zweekhorst M, Miranda-Galarza B, van Brakel W, Irwanto, et al. Learning from a leprosy project in Indonesia: making mindsets explicit for stigma reduction.

Development in Practice 2015; 25(8):1105-1119 DOI: [10.1080/09614524.2015.1081155](http://dx.doi.org/10.1080/09614524.2015.1081155)

1. Reid H, Kibona S, Rodney A, McPherson B, Sindato C, Malele I, et al. Assessment of the burden of human African trypanosomiasis by rapid participatory appraisal in three high-risk villages in Urambo District, Northwest Tanzania. [Afr Health Sci.](https://www.ncbi.nlm.nih.gov/pmc/articles/PMC3462543/) 2012 Jun; 12(2): 104–113. doi: [10.4314/ahs.v12i2.5](https://dx.doi.org/10.4314%2Fahs.v12i2.5)
2. Sahota RS, Sanha S, Last A, Cassama E, Goncalves A, Kelly AH, et al. Acceptability and perceived utility of different diagnostic tests and sample types for trachoma surveillance in the Bijagos Islands, Guinea Bissau. Trans R Soc Trop Med Hyg 2021; 115(8): 847–853,

DOI: [10.1093/trstmh/traa179](https://doi.org/10.1093/trstmh/traa179)

1. Waiswa C, Azuba R, Makeba J, Waiswa IC, Wangoola RM. Experiences of the one-health approach by the Uganda Trypanosomiasis Control Council and its secretariat in the control of zoonotic sleeping sickness in Uganda. Parasite Epidemiol Control. 2020;11:e00185. doi: 10.1016/j.parepi.2020.e00185.
