# Supplementary material for: The transdisciplinary research process and participatory research approaches used in the field of neglected tropical diseases: A scoping review
Source: PLoS Negl Trop Dis. 2025 Apr 1;19(4):e0012959. doi: 10.1371/journal.pntd.0012959 (PMC11977956; doi:10.1371/journal.pntd.0012959)
Supplement: S4 Appendix — (DOCX) [file pntd.0012959.s004.docx]

**S4 Appendix**

A template data extraction instrument

This template outlines the key study characteristics and participant engagement activities. It includes study objectives, stakeholder involvement and key aspects of intervention, implementation and evaluation.

| Details and Characteristics of the studies:  Authors' names,  Year of publication,  Countries of origin,  Objectives of the studies,  Concept,  Context,  Participants/stakeholders, and  Sample size    Characteristics of participant engagement activities:  Problem identification/prioritisation/integration,  Joint agenda and implementation,  Review of pilot project/interventions,  Limitations and barriers, and  Facilitators |
| --- |
