# Supplementary material for: The transdisciplinary research process and participatory research approaches used in the field of neglected tropical diseases: A scoping review
Source: PLoS Negl Trop Dis. 2025 Apr 1;19(4):e0012959. doi: 10.1371/journal.pntd.0012959 (PMC11977956; doi:10.1371/journal.pntd.0012959)
Supplement: S5 Appendix — (DOCX) [file pntd.0012959.s005.docx]

**S5 Appendix**

**Characteristics of the included studies**

This appendix provides a detailed summary of the characteristics of the included studies, organised in a structured data extraction sheet. It includes information on study authors, publication year, country of study, objectives, research concepts, context, sample size, and key findings. The table categorises studies based on their approach to stakeholder engagement, problem identification, intervention implementation, and project review. Additionally, it highlights study limitations, barriers to intervention success, and key facilitators that contributed to effective program outcomes.

| Articles  (Authors, Year of Publication) | Country | Objectives | Concept | Context | Sample size (SS) Participants/  Stakeholders (SH) | Problem  Identification,  prioritisation and integration | Joint Agenda  And Implementation | Review of Project/  Interventions | Limitations and Barriers | Facilitators |
| --- | --- | --- | --- | --- | --- | --- | --- | --- | --- | --- |
| Ackumey MM, Kwakye-Maclean C, Ampadu EO, de Savigny D, Weiss MG. 2011.  (1) | Ghana | Increase access to BU treatment and improve early case detection and case management. | Participatory Approach  Using mixed methods included a review of patients’ records, a review of program  reports, a stakeholder forum, key informant interviews, focus group discussions, clinic visits and observations. | Rural and urban  (Hospital-based and community clinics) | SH forum n=35 Records reviewed n=297  1. The programme manager of the National BU control programme,  2. The municipal chief executive of the Ga-West municipality,  3. Some municipal health staff,  4. BU Prevention and Treatment staff of World Vision Ghana,  5. Hospital doctors and nurses,  6. Officials from Ghana education service,  7. Treated or discharged patients, carers of child patients  8. Community-Based Surveillance Volunteers,  9. Municipal environmental health officers | 1. A stakeholder forum reviewed the BUPaT programme activities,  2. Explored issues regarding health services delivery,  3. The capacity of health staff to deliver BU-related services,  4. The integration of programme activities in communities and schools,  5. Key informant interviews and FGDs with community members, to look at access to care, successes, and challenges.  6. Documents and patient records review,  7. On-site clinic visits to assess the effectiveness of decentralising treatment | 1. Compilation of patient database - health staff trained to record patient information using WHO forms, disease outcomes, and clinical/surgical procedures,2. Improving access to treatment by providing incentives to surgeons, providing food and transport for patients, 3. Training of health staff and other SH  4. Equip existing clinics to serve as treatment centres for wound care and antibiotic treatment and staff training,  5. Health education and community surveillance activities to increase awareness, improve case detection and encourage early reporting. | 1. The early case detection and treatment indicate programme success in managing BU,  the increasing number of patients receiving treatment at the hospital,  2. Resulted in improved collaboration among stakeholders,  3. Improved community-based surveillance and awareness of BU,  4. improved access to treatment - including antibiotic therapy (good outcomes) besides surgery and wound care,  5. included private healthcare providers in the health system to increase access to antibiotic therapy. | 1. The delay in seeking treatment,  2. Limited health budget,  3. Lack of infrastructure in municipal health centres to perform surgery,  4. Lack of ward space to admit affected persons for surgery.  5. Fear of amputations, loss of livelihood and long absence of caregiver from home  6. Misinformation by traditional healers  7. Difficulty in differentiating BU from other causes. | 1. Collaboration and networking among stakeholders, including their training, strengthen the governance sub-system and improve the health delivery of the programme  2. Using WHO-recommended antibiotics improved treatment outcomes and reduced expensive surgery. |
| Apte H, Chitale M, Das S, Manglani PR, Mieras LF. 2019.  (2) | India | 1. Accept contact screening  2. Single dose  rifampicin as chemoprophylaxis for leprosy in  Dadra and Nagar Haveli, India | Participatory Approach Mixed methods - Qualitative, cross-sectional, and quantitative study | Rural setting | SS total n=111  (SSIs n=62  FGDs n=49)  1. Index patients n=15  2. Contacts of the index patients n=52  3. Key informants in the community n=9  4. Health workers responsible for implementing the SDR-PEP intervention n=25  5. Project supervisors and research assistants n=10 | 1. Interviews and FGDs were conducted, and they found that contact screening and SDR-PEP administration as  chemoprophylaxis for leprosy was very well accepted by the main stakeholders and their contacts,  2. They were confident about the information they received and understood the reasons for intervention. | 1. Assess the acceptability of contact screening  and SDR-PEP administration by the stakeholders involved.  2. Increased coverage or mass distribution of SDR-PEP helps overcome the problem of index patients not wanting to disclose their status to contacts outside the household. | 1. SDR-PEP intervention is seen as beneficial for contacts of leprosy patients by all participants.  2. Should always be mindful of the potential negative risks of disclosing leprosy status.  3. A gender-sensitive approach for contact screening is  very important, and 4. An adapted system ensures  the participation of societal groups with specific sociocultural traditions and behaviours related to health interventions. | 1. Health workers were involved in data collection by  helping the researchers to get access to the families and to translate when needed. Difficult for outsiders to locate the participants without  assistance from someone who knows the area.  2. The high-level managers were not included in the study.  3. No FGD  was conducted with persons that had refused to take SDR-PEP.  4. FGDs could not be organised for both sexes in all categories. | 1. Fear of disclosure of having leprosy has not been a barrier to the  introduction of chemoprophylaxis in DNH, and 2. It supports the  implementation of the national guidelines for post-exposure to leprosy. |
| Awah PK, Boock AU, Mou F, Koin JT, Anye EM, Noumen D, et al. 2018  (3) | Cameroon | Developing a Buruli ulcer (BU) community of practice in Bankim, Cameroon | Participatory approach | Rural setting | SS n=212  1. Anthropologists n=3,  2. Government Officials n=5,  3. NGOs,  4. Current Patients n=77  5. Ex-patients n=31,  6. Hospital/health staff n=24,  7. Community health workers (CHWs) n=41,  8. Traditional healers n=31. | Workshop, meetings and participant observation, and interviews  1. To develop a feasible package of proposed interventions to enhance BU outreach, and  2. Establish a BU community of practice. | 1. Raise community awareness about BU,  2. Give proactive roles and mobilise CHW and traditional healers, 3. Provide transport, lodging, and food to patients and their caretakers if necessary  4. upgrade treatment facility at Bankim | 1. Conduct outcome, process, and impact evaluations to assess the effectiveness of the intervention in terms of BU detection, treatment adherence,  2. BU Community of Practice stakeholder collaboration promotes trust between health centre staff and community stakeholders. | 1. Cultural perceptions of why wounds do not heal in a timely manner; 2. Fear of hospital treatment and trust in hospital staff, and  3. Pragmatic issues such as  i) Poor transportation, ii) Housing,  iii) The availability of food for patients and caretakers when treatment requires hospitalisation | 1. Establishing close ties to community stakeholders constitutes an essential part of the health system strengthening  2. establishing trust and lines of communication with community leaders enables swift action and increased opportunities for local problem-solving |
| Beran D, Lazo-Porras M, Cardenas MK, Chappuis F, Damasceno A, Jha N, et al. 2018.  (4) | Mozambique, Nepal, and Peru | The co-creation of interventions for NCDs and NTD: insights from a community health system project.  (The COmmunity HEalth System InnovatiON  (COHESION) Project) | Participatory Approach  Mixed-methods- Community mapping, In-depth interviews  FGDs,  Observations, secondary data collection. | Rural and urban settings | Across different actors: Nepal n=113 - 231  Peru n= 10-84  Mozambique n=6-11  1. Community medicine physicians,  2. Public healthcare workers,  3. Local community members in Nepal, Peru, and Mozambique to obtain their perspectives | 1. Multiple Stakeholder meetings at policy, health system, and community levels to provide information on selected diseases, 2. Results were presented, and participants were asked for suggestions to improve the situation of people affected by NCDs or NTDs,  3. Aimed to strengthen the health systems | 1. Advocacy for NCDs and NTDs at the policy level.  2. Better diagnostic tools for early detection at primary healthcare (PHC),  2. Media campaigns, posters, and educators on NTDs in the community,  3. Strengthening of PHC, peer support, women's groups,  4. Training health personnel – nurses, lab, and pharmacists, focusing on the treatment and management of diseases. | 1. Adapt the tools and methods used for co-creation to the characteristics of the stakeholders and each setting,  2. Analyse the implementation of the intervention (co-created by the stakeholders based on the best available evidence)- whether quantitatively or qualitatively or using the tools. | 1. No guarantee of funding to implement the interventions.  2. The need for investment in time and not knowing which interventions may result.  3. Issues about co-creation and how to involve everyone's views when developing interventions. | This approach allows an opportunity to develop interventions with local stakeholders and ensure they all agree to the implementation of interventions. |
| Degeling C, Brookes V, Lea T, Ward M. 2018.  (5) | Australia | 1. Helping communities potentially at risk of rabies prepare for an incursion,  2. Develop strategies to manage a rabies outbreak. | One Health Participatory Approach  Mixed-methods  InterviewsCollaborative workshops in study sites  Review of policy documents, by-laws, and grey literature | Rural and urban settings:  Three sites  1. East Arhem (EA)  2. North Peninsular area (NPA)  3. Cairns | Informal SSIs n=28 before the study, n=23 months before and n=5 after the study period  5 Collaborative storyboard workshops  EA n=22  NPA n=26  Cairns n=13  1. Indigenous communities (local residents and traditional elders and councillors) n=5,  2. Teachers, human health professionals, veterinarians n=7,  3. Rangers and animal control workers n=6,  4. Local residents n=10. The subjects in Cairns were non-indigenous. | 1. Interviews  2. Collaborative workshops to discuss topics that were important to establishing the rabies surveillance programmes  3. Using storyboard methodology to plan measures to control rabies in case of an outbreak | Policy background:  AUSVETPLAN  Based on One Health, disease preparedness:  1. Community education,  2. Accurate dog census,  3. Case identification,  4. Contact tracing and quarantine.  5. Vaccination,  6. Controls on dog movements and monitoring  7. Cultural beliefs did not support the culling of dogs  8. Help owners manage their own dogs during outbreak | Evaluate participants’ acceptance:  1. A mass vaccination program for dog populations in the event of a rabies outbreak,  2. Restricting canine movements, which most would comply with if they understood why it was necessary but focus on helping them manage their dogs,  3. Trap-vaccinate-release programme acceptable for the dingo population. | 1. Serious concerns if the indigenous communities will not be able to comply with AUSVETPLAN,  2. Dogs and dingoes can have significant cultural and social importance,  3. The indigenous people have poor infrastructure, 4. Lack of information  5. Dominant  cultural norms  6. Food security concerns. | 1. Must listen to and respect sociocultural differences.  2. Understand their concerns, and educate them and attend to their infrastructure needs |
| El Katsha S, Watts S, Khairy A, El-Sebaie O. 1993.  (6) | Egypt | Community participation for Schistosomia-  sis control in Egypt | Participatory Approach  Qualitative study: IDIs and FGDs  Community mapping  Baseline census of villagers,  Observation sessions of water contact activities | Rural settings | SS Local staff n=12  Local government staff, including  1. physicians n=2  2. lab technicians  n=4  3. snail specialists, n=3  4. other workers n=3  health personnel, primary school teachers, and staff concerned with irrigation, water, and sanitation. | Meetings were held between researchers, staff members, and villagers to get  1. A deeper understanding of why exposure to canal water persists,  2. What could be done to minimise this contact? | Health workers and villagers are more aware for:  1. community education,  2. Testing,  3. Treatment of villagers and school children if tested positive.  4. An improvement in the clinic recording system acted as an incentive for  clinic staff to provide and record tests and give treatment. | Analyse the interventions:  1. Upgrade facilities  2. Improve water and sanitation by preventing contamination,  3. Improve health education and outreach, using multimedia - priority to primary school children,  4. Improve lab facilities,  5. Increased testing, leading to better compliance to get treated,  6. Staff training,  7. Record keeping,  8. Environmental control of snail population. | 1. A lack of integration of the activities of the various staff  responsible for schistosomiasis-related activities,  2. Difficult to reach villagers and inform them about schistosomiasis,  3. No one is in charge of village-level education. | 1. The ultimate aim of the project is to identify  feasible, sustainable interventions utilising local skills and knowledge.  2. The collaboration has improved local health services and treatment and increased knowledge and awareness in the communities. |
| Freudenthal S, Ahlberg BM, Mtweve S, Nyindo P, Poggensee G, Krantz I. 2006.  (7) | Tanzania | Initiating a school-based participation action research in the prevention of Schistosomiasis in Northern Tanzania | Participatory Action Research  Organised activities, surveys | Rural setting | SS noted, many groups involved  1. Village leaders  2. Medical health professionals - Tanzanian team:  i) One medical doctor,  ii) Several community health nurses,  iii) Laboratory technicians,  3. Social scientists from Tanzania and two European countries,  4. Teachers and pupils. | 1. 2 workshops were held at the hospital,  2. School activities were initiated by the screening of  Schoolchildren,  Devt of slogans, videos of drama, songs and dances  3. Teachers and pupils were involved with a household sanitation survey,  4. Data was analysed and discussed to stop the infections or to sustain the reduction. | SH are involved in organised activities  1. The researchers and community nurses screened schoolchildren n=1146 and treated all the infected children.  2. The engagement of teachers and pupils can spearhead changes as described by WHO, given that the school settings represent a complex landscape of actors with diverse interests and social locations.  3. Teachers created new school curriculum | 1. Evaluate the sustainability  of the prevention efforts as they are dependent on  working within existing community structures-teachers, pupils, village elders, and community members rather  than creating new ones,  2. Screening improved communication and collaboration,  3. Involvement of school children and teachers to generate knowledge. | Health education programmes related to schistosomiasis control are not sustainable unless they are integrated into the general  health services. | The success of this project is dependent on community participation. |
| Gautam V, Bhardwaj P, Saxena D, Kumar N, S D. 2020.  (8) | India | Multisectoral approach to achieve canine rabies control zone using Intervention Mapping | Participatory Approach  An exploratory, cross-sectional study  With Intervention mapping | A hospital complex, a medical Institute building, and a residential complex in urban Jodhpur | SS n=53  1. Children above 10 years old of faculty and staff residing on the campus n=8  2. Residents of the Institute n=13  3. Undergraduate and Postgraduate students n=12  4. Security staff n=12  5. Municipal cooperation workers n=2  6. NGOs involved in vaccine procurement (private sector) n=2  7. Members from the organisation involved in the stray dog handling n=2  8. Members of State Health Authorities n=2 | A discussion was carried out:  1. To explore the challenges faced due to the campus stray dog population,  2. To initiate a dialogue among the residents to reach a mutual consensus regarding measures to be adopted to design a canine rabies-controlled zone, 3. Developing a comprehensive dog-mediated rabies prevention framework to prevent rabies in stray dogs. | 1. Canine rabies vaccination,  2. Prevention of provoked dog bites, 3. Campus stray dog population control - focus on collaboration between community members, the veterinary sector, and NGOs  4. Supply of canine rabies vaccine,  5. Conduct sterilization activity for dogs (by providing Ketamine Hydrochloride). | Annual evaluation: 1. Effectiveness of the Community sensitization activity: Health education session on dog behaviour, during an encounter with an unknown stray dog for children, residents on the campus, and security staff.  2. Creating a Campus stray dog welfare committee with responsible feeding, vaccination, and sterilisation. | 1. Findings were from a single institution with a limited number of stray dogs.  2. The efforts of stakeholders in rabies control were  fragmented. | Intervention Mapping was utilized to develop the intervention model, which is an evidence-based method that guides the development of the program holistically |
| T Jaeggi, P Manickam, MG Weiss, MD Gupte. 2012  (9) | India | Stakeholders' perspectives on perceived needs and priorities for leprosy control and care | Participatory Approach Qualitative study  IDIs | Rural and Urban settings | SS n=17  1. Six leprosy patients (2 recently diagnosed, 2 completed treatment, 2 with disability),  2. Four experts (incl. programme manager and Deputy Director of NGO Leprosy Hospital and 2 experts-state/global),  3. Five health workers at the primary healthcare centre (1 Medical officer, 1 health inspector, 1 pharmacist, and 2 village health nurses),  4. Two local community leaders. | Qualitative in-depth interviews, guided by an agenda, covering  i) perceived epidemiologic trends,  ii) the status of control and the quality of patient care,  iii) stress more on patient care, control, and care with healthcare staff. | 1. Patients concerned with access to services, 2. Healthcare providers focused on health system support (personnel, surgical backup, and availability of supplies and medicines).  3. Policymakers focused on programme operations and trends concerning epidemiology and the status of elimination and tools for control.  4. The community leaders were relatively less engaged. Community awareness requires the dissemination of information about leprosy through different media. | 1. The integration of leprosy services in primary health care; cases are detected based on clinical symptomatology during regular health programme operations.  2. Has resulted in improved access to outpatient treatment and home care for the rural population,  3. Less costly for services and  4. Less time to obtain needed treatment.  Healthcare personnel and expert policymakers agreed that this approach was crucial for maintaining sustainable leprosy services. | 1. Longer waiting times at the health centre and less available home care.  2. The political commitment to control leprosy has lessened over the years.  3. The respondents  were fewer in number and were purposively  selected. This may limit the external validity (especially the group of patients, healthcare  providers, and community leaders). | An integrated approach for leprosy services in primary care and the input of stakeholders are needed to guide a more effective approach to treating leprosy patients to reduce the burden of disease in the population. |
| Kuipers P, Joy A, John A, Raju MS. 2018.  (10) | India | Stakeholder perspectives on  preventing a delayed diagnosis of leprosy | Qualitative and participatory approach | Urban settings | SS n=92  1. IDIs n=39  i) People with leprosy n=35  ii) community n=4  2. FGDs n=30  i) people affected by leprosy,  ii) family and community  members of leprosy-affected people,  iii) community-level service providers  3. Research Translation Groups n=23:  i) Key service providers and senior staff of The Leprosy Mission Trust, India n=10  ii) Chhattisgarh District Leprosy Officers and associated staff n=13 | 62 suggestions to address the delayed diagnosis of leprosy are the perspectives of a number of key stakeholders with personal, relevant practical experience of this issue.  They were  1. prioritised,  2. ranked, and  3. recommended the greatest investment in community education and awareness. | 1. Prioritising community-level education, community awareness, and community-level actions and basic skills.  2. Improving service and systems, and policy focus  3. focusing on remote areas where leprosy is endemic | To evaluate its success, we must assess the following:  1. The skills and roles of people at the community level  2. Greater skilling of grassroots health workers at primary healthcare centres  3. A broad dissemination approach to education and awareness via TV and media, including schools  4. Targeted and active case-finding approaches and incentives for workers to identify potential patients will assist in early diagnosis | 1. The findings are likely to have had a slightly different emphasis had we chosen different  participants.  2. The participants were purposively  chosen and was not a representative sample of people affected by leprosy.  3. The research assts were men and this would have impacted information sharing by women participants. | 1. Potential action to promote early detection will acknowledge that  1. a holistic response will entail multiple actors, and  2. Strong experience in community engagement |
| Means AR, Jacobson J, Mosher AW, Walson JL. 2016.  (11) | Not stated but in Sub-Saharan Africa  (according to IRB stipulations) | A qualitative research approach to integrated healthcare delivery for NTD programmes | Participatory Approach  A cross-sectional qualitative research study | Low resource settings -Rural | SS n=41  NTD stakeholder groups n=7  1. Multilateral Organisation n=2  2. Funding Partner Agencies n=2  3. Implement Partners n=4  4. National MoH workers n=5  5. District MoH health workers n=6  6. Community drug distributor n=8  7. Community members n=14 | Semi-structured key informant interviews were used with a mix of respondent and informant-style questions. | 1. Significant variations in definitions,  2. The differential effectiveness of specific activities, when integrated,  3. The influence of integration on community member engagement,  4. The influence of funders on integrated programming,  5. Facilitators  6. Barriers to effective integration, and  7. The effects of  integration on health system strengthening.  Three types of integration:  1. Structural  2. Process  3. Technical | 1. Evaluation of the efficiencies in time and human resources,  2. Increased uptake in services through integrated programming,  3. Ability to share elimination lessons learned across disease initiatives, 4. Leadership structures that promote communication between disease-focal persons.  5. Public health stakeholders should embrace a broader perspective of community-based health needs, including and beyond NTDs, and available platforms for addressing those needs. | 1. Responses may have been biased if interviewed individuals felt that their feedback might reach employers or community leaders.  2. A second limitation is that the data analysis did not involve multiple coders; thus, intercoder reliability was impossible to establish.  3. Lastly, the stakeholders’ views do not represent all stakeholders. | 1. This study highlighted the stakeholder perceptions of the complex process of NTD integration.  2. Stakeholders should standardize and redesign their reporting systems to capture information regarding which NTD program activities are integrated with other activities and to share the data promptly |
| Onasanya A, Keshinro M, Oladepo O, Van Engelen J, Diehl JC. 2020.  (12) | Nigeria | A stakeholder analysis of  schistosomiasis - Diagnostic co-creation in South-West Nigeria | Participatory action research,  A case study approach  Qualitative: Key-informant interviews, IDIs, FGDs,  expert recommendations and  Quantitative:  document analysis | Rural and agricultural settings | 36 SH were identified, but 33 interviewed as some unavail  1. Government (State Disease Surveillance and Notification Officer and Federal NTD officer) (n=2),  2. Parents or guardians of children with schistosomiasis (n=5)  3. Health sector- experts from public health and clinical medicine and private sector (n=17),  4. Community and religious leaders and mobiliser (n=3),  5. Teachers (n=2),  6. NGOs (n=1),  7. Traditional healer (n=1),  8. Academia (n=3),  9. Financing (n=1) | 1. A qualitative study (In-depth and Key informant) interviews and Focus Group Discussions (FGD) with SH.  2. Stakeholders were analysed and ranked based on their power and interest | 1. This study assessed and mapped stakeholders' interest, influence/power, and position within the schistosomiasis  diagnostics landscape concerning the development of a device for improved diagnosis of schistosomiasis.  2. Engaging and co-  creating with stakeholders in diagnostic device development and  adoption is important for the successful deployment and use of diagnostic devices. | 1. Evaluate how each stakeholder may be engaged for co-creation and device usage.  2. The key players within the health system (medical and organizational) and policy environment are essential for device co-creation and validation, strategizing, and guiding product development.  3. The donor has the highest level of power for co-creation, and they determine the direction of the health policy. | 1. One limitation of the study was that some stakeholders, for instance, political actors and media, were not interviewed  2. The device development has a long life cycle.  3. Some findings may not be generalizable to other parts of the country.  4. The culture of the predominant ethnic group can affect how stakeholders interact with each other and the power dynamics. | To ensure that diagnostic devices are useful in the context for  which they are created, it is critical to involve end-users and  other important stakeholders throughout the entire co-creation  process. |
| Ozano K, Dean L, Adekeye O, Bettee AK, Dixon R, Gideon NU, et al. 2020.  (13) | Liberia and Nigeria | Multicountry learnings  to strengthen health systems by connecting and co-creating potentially sustainable solutions to implementation challenges and health programmes within the NTD programmes in Liberia and Nigeria | Participatory Action Research (PAR) | Rural and urban settings | Interviews n=20 (Liberia 5, Nigeria 15)  1. Ministries of Health (National and sub-national),  2. Local Government  3. Frontline health staff - Volunteer community drug distributors,  4. Communities - people living or working in NTD communities,  5. Academia and Research Institutions  6. Donors,  7. NGOs and Implementing partners,  8. Other Development partners. | 1. Reflections from research partners at all levels were collected and analysed using PAR principles as a quality and ethical standards framework to identify new principles that may be more aligned with Health Systems Strengthening (HSS).  2. The multi-disciplinary  background with expertise in both HSS and PAR approaches added trustworthiness and allowed for cross-validation of new principles, adding quality and relational dynamics within PAR and HSS. | Five Principles and the potential quality outcomes for health systems:  1. Recognize communities as units of identity and define stakeholder participation,  2. Enable flexible action planning that builds on existing structures,  3. Address health systems and research power differentials,  4. Embed relational practices that lead to new political forms of participation and inquiry within health systems,  5. Develop structures for ongoing learning at  multiple levels of the health system. | Explore:  1. Health System relational dynamics,  2. The processes that support systems and policy change, and  3. Some barriers and enablers that facilitate ethical, democratic Health Systems Strengthening overall.  4. This research has drawn on multi-country findings to develop five guiding principles for ethical standards, quality, and ongoing learning in implementation research utilizing a PAR framework and strengthening health systems. | 1. The lack of inclusion of  community-level stakeholder  perspectives.  2. Liberia has a complex socio-political history with disruptions in its NTD services.  3. Nigeria is culturally diverse and multi-ethnic and has ongoing community-directed treatment for NTDs. | 1. The application of local knowledge in this research allows for shared  learnings from two diverse contexts that could be useful in guiding other programmes aiming to strengthen health systems and develop  sustainable programmatic improvements.  2. Understanding best practices in navigating issues of power and participation is needed to establish equitable,  quality and sustainable partnerships for PAR. |
| Peters R, Lusli M, Zweekhorst M, Miranda-Galarza B, van Brakel W, Irwanto, et al. 2015.  (14) | Indonesia (Cirebon district) | The importance of mindsets to assess the effect of a contact intervention in reducing leprosy-related stigma in Indonesia | Interactive Learning and Action (ILA) methodology,  Mixed-methods Interviews FGDs, meetings, workshops, and reflections quantitative study | Rural and urban settings | IDIs n=53  20 FGDs  1. The team consisted of three PhD students, one  post-doctoral researcher, four senior researchers, and two professors  2. MoH-health professionals, academics, Provincial Health Office, District Health Office, and the Puskesmas, health service volunteers, NGOs, disabled people's organisations, religious leaders, community/village leaders, local teachers, micro-credit Organisations, leprosy-affected people, and the public, and other key informants in the field of disability and leprosy. | 1. Research Team met with key stakeholders and other key informants. Workshops, dialogues, informal meetings, and FGDs were conducted among relevant stakeholders.  2. Paired interventions  were employed for ethical reasons: counselling and contact, socio-economic development and counselling, and contact and socio-economic development.  3. The stakeholders also decided on an in-depth study to understand leprosy and stigma in the local context. | 1. The three paired interventions addressed different  levels of stigma and were multi-faceted: Counselling and contact, Socioecon devt and counselling, and contact and Sociecon devt.  2. The participation and empowerment of people affected by leprosy focused on rights and aimed to increase knowledge. | 1. The differences in mindsets should be viewed through a more positive lens because they provide feedback  and represent windows of opportunity for learning.  2. Being aware of power issues and finding ways to achieve equity.  3. It is sometimes important to  defend key principles and discontinue or adapt collaboration to establish stronger relationships.  4. Willingness and flexibility of the research approach helps to build relationships. | 1. Stakeholders had different mindsets with differences in aspirations, attitudes to scientific validity  and ethics of research,  2. Different expectations in terms of interventions and timing, conflicting  intrinsic models of disability, and diverse attitudes to people affected by leprosy. | 1. The involvement of appropriate stakeholders in the form of people and organisations; an awareness  of power dynamics; the development of relationships within a team and between the stakeholders;  2. Appropriate structures for learning and reflection will lead to better interventions and implementation. |
| Reid H, Kibona S, Rodney A, McPherson B, Sindato C, Malele I, et al. 2012.  (15) | Tanzania | The burden of human African trypanosomiasis (HAT) in Urambo District, Northwest Tanzania | A rapid participatory appraisal (RPA)  qualitative and quantitative methods | Rural settings | SS n=38  Higher-level:  1. National Institute of Medical Research Laboratory technicians and research scientists n=3  2. Ministry of Health informants n=2  3. District Council Development officers n=1  4. Ministry of Livestock Development and Fisheries n=2  5. Kaliua Health Centre staff n=2  Village level:  1. Village Chairperson and Executive officer n=4  2. Village Influential Women n=3  3. Case studies n=6  4. High-risk individuals n=6  5. General population n=4  6. Sukuma tribe herdsman n=1  7. Traditional healer n=2  8. Dispensary worker n=1  9. Chairperson of Tseste control committee n=1 | Semi-structured Key informant Interviews.  1. Participatory tools were used to investigate how villages prioritise health issues affecting their lives.  2. A listing and ranking exercise was conducted. | 1. HAT creates a significant burden in terms of disease morbidity and mortality experienced by the patient and social and economic costs to patients and their families.  2. Pair-wise ranking confirmed the importance of both HAT and malaria at the village level.  3. There is a need for better reporting of disease, earlier diagnosis, and treatment at the local level; a microscope will help diagnose malaria and free travel for patients to the hospital for early diagnosis. | 1. Designing appropriate risk reduction strategies with community stakeholders would facilitate efficient and effective program design.  2. The utility and acceptability of various alternative approaches like permethrin-impregnated clothing; using the most cost-effective and efficient visual bait tsetse traps; ensuring the most appropriate placement of visual baits; and the role of periodic aerial insecticide spraying. | 1. It is extremely difficult to diagnose HAT clinically because first-stage symptoms are indistinguishable from other more common febrile illnesses such as malaria.  2. Furthermore, the neurological symptoms of second-stage HAT may be confused with cerebral malaria or tuberculosis  3. Asking villagers to reduce time  spent in and around heavily tsetse-infested forest areas  is unlikely to be feasible given the communities' critical  dependence on the land for their largely subsistence  economy. | Adopting a participatory mixed methods approach provided an efficient means to investigate the impact of this neglected disease in an isolated, resource-limited context and provided valuable results to inform future policy and operations research priorities |
| Sahota RS, Sanha S, Last A, Cassama E, Goncalves A, Kelly AH, et al. 2021.  (16) | Guinea Bissau | Acceptability and perceived utility of different diagnostic tests  and sample types for trachoma surveillance in the Bijagos Islands, Guinea Bissau. | Participatory approach- SSIs and FGDs | Remote, rural setting | SH SSIs n=5  42 community SSIs across 9 communities n=21  6 FGDs across 3 communities  A stakeholder mapping exercise  1. One ophthalmic doctor,  2. One ophthalmic nurse,  3. One public health doctor  4. Two NGO representatives  5. The community leader and other community members. | Semi-structured interviews (SSIs) were held with  1. community members in Bubaque, the island in the Bijagos  archipelago with the largest population and  2. key stakeholders  working on the Guinea Bissau trachoma programme.  The SSIs were followed by FGDs with community members in Bubaque. | Community participants and stakeholders perceived the benefits of laboratory testing, including use of machines, over clinical  examination and would like these alternative indicators for trachoma diagnosis to become feasible at scale. | 1. The community members and stakeholders preferred modern laboratory-based diagnostic techniques.  2. Thus, more emphasis was placed on test accuracy than the speed of the result.  3. The integration of laboratory tests into the national health systems is needed, along with investment in resources, personnel, and infrastructure  4. Foreign Drs were perceived to be more experienced and knowledgeable, but would not stay for long. | 1. The community members were invited by the leader, which may have led to biased results,  2. A field translator was needed to ask appropriate questions,  3. Participants interviewed from Bijagos Island are culturally different from the other islands, hence,  Local geographical representativeness is also limited  4. Results lacked a global perspective as international stakeholders were not contactable during data collection. | 1. The views of  community members and key stakeholders involved in the trachoma elimination programme were explored. 2. Further-  more, community member perspectives were explored via both  SSIs and FGDs, enabling the triangulation of results. |
| Waiswa C, Azuba R, Makeba J, Waiswa IC, Wangoola RM. 2020.  (17) | Uganda | The Uganda Trypanosomiasis Control Council (UTCC) to  handle all issues of tsetse and trypanosomiasis control in Uganda.  Strengthening of UTCC platform using One Health | One Health Approach – involves cattle and humans,  Interviews with local govt, community members and livestock owners | Rural | SS size noted 278 human subjects in study area  1. The UTCC (veterinary, medical, agriculture, environment, wildlife, lands, and political science),  2. Vector control division of MoH (medical specialist and host of Manager of the Uganda National sleeping sickness control program),  3. Leaders in district and sub-counties (technical; vet, medical, entomology, social work, and politicians)  4. Community | The Rapid Impact and Making a Difference project of Tackling Infections to Benefit Africa (TIBA) Uganda interventions offered the opportunity of  1. Capturing experiences of the multi-stakeholder engagements and community meetings. Including cattle and human studies. | 1. Rapid diagnostics with a common platform are essential to enable rural and growing city populations.  2. It is essential to find cases quickly to continue the path to elimination. | It is very crucial to develop one health diagnostic facilities that can deliver rapid results to the populations that need them for rapid interventions to take place | The community was concerned about the inconsistent supply of chemicals by the private sector for animal intervention (Tsetse and tick control). Uganda had tick resistance to some of the products | Stakeholders involved in applying a One health approach to control zoonotic sleeping sickness across the most important active human African trypanosomiasis focus in East Africa. |

References:

1. Ackumey MM, Kwakye-Maclean C, Ampadu EO, de Savigny D, Weiss MG. Health services for Buruli ulcer control: lessons from a field study in Ghana. PLoS Negl Trop Dis. 2011 Jun;5(6):e1187.

2. Apte H, Chitale M, Das S, Manglani PR, Mieras LF. Acceptability of contact screening and single dose rifampicin as chemoprophylaxis for leprosy in Dadra and Nagar Haveli, India. Leprosy Review. 2019;90(1):31–45.

3. Awah PK, Boock AU, Mou F, Koin JT, Anye EM, Noumen D, et al. Developing a Buruli ulcer community of practice in Bankim, Cameroon: A model for Buruli ulcer outreach in Africa. PLoS Negl Trop Dis. 2018 Mar;12(3):e0006238.

4. Beran D, Lazo-Porras M, Cardenas MK, Chappuis F, Damasceno A, Jha N, et al. Moving from formative research to co-creation of interventions: insights from a community health system project in Mozambique, Nepal and Peru. BMJ Glob Health. 2018;3(6):e001183.

5. Degeling C, Brookes V, Lea T, Ward M. Rabies response, One Health and more-than-human considerations in Indigenous communities in northern Australia. Soc Sci Med. 2018 Sep;212:60–7.

6. El Katsha S, Watts S, Khairy A, El-Sebaie O. Community participation for schistosomiasis control: a participatory research project in egypt. Int Q Community Health Educ. 1993 Jan 1;14(3):245–56.

7. Freudenthal S, Ahlberg BM, Mtweve S, Nyindo P, Poggensee G, Krantz I. School-based prevention of schistosomiasis: initiating a participatory action research project in northern Tanzania. Acta Trop. 2006 Nov;100(1–2):79–87.

8. Gautam V, Bhardwaj P, Saxena D, Kumar N, S D. Multisectoral approach to achieve canine rabies controlled zone using Intervention Mapping: Preliminary results. PLoS One. 2020;15(12):e0242937.

9. Jaeggi T, Manickam P, Weiss MG, Gupte MD. Stakeholders perspectives on perceived needs and priorities for leprosy control and care, Tamil Nadu, India. Indian J Lepr. 2012 Sep;84(3):177–84.

10. Kuipers P, Joy A, John A, Raju MS. A pilot study using participatory, translational, social science research methods to explore stakeholder perspectives on preventing delayed diagnosis in leprosy. Leprosy Review. 2018;89(2):124–38.

11. Means AR, Jacobson J, Mosher AW, Walson JL. Integrated Healthcare Delivery: A Qualitative Research Approach to Identifying and Harmonizing Perspectives of Integrated Neglected Tropical Disease Programs. PLoS Negl Trop Dis. 2016 Oct;10(10):e0005085.

12. Onasanya A, Keshinro M, Oladepo O, Van Engelen J, Diehl JC. A Stakeholder Analysis of Schistosomiasis Diagnostic Landscape in South-West Nigeria: Insights for Diagnostics Co-creation. Front Public Health. 2020 Oct 30;8:564381.

13. Ozano K, Dean L, Adekeye O, Bettee AK, Dixon R, Gideon NU, et al. Guiding principles for quality, ethical standards and ongoing learning in implementation research: multicountry learnings from participatory action research to strengthen health systems. Health Policy Plan. 2020 Nov 1;35(Supplement_2):ii137–49.

14. Peters R, Lusli M, Zweekhorst M, Miranda-Galarza B, van Brakel W, Irwanto, et al. Learning from a leprosy project in Indonesia: making mindsets explicit for stigma reduction. DEVELOPMENT IN PRACTICE. 2015 Nov 17;25(8):1105–19.

15. Reid H, Kibona S, Rodney A, McPherson B, Sindato C, Malele I, et al. Assessment of the burden of human African trypanosomiasis by rapid participatory appraisal in three high-risk villages in Urambo District, Northwest Tanzania. Afr Health Sci. 2012 Jun;12(2):104–13.

16. Sahota RS, Sanha S, Last A, Cassama E, Goncalves A, Kelly AH, et al. Acceptability and perceived utility of different diagnostic tests and sample types for trachoma surveillance in the Bijagos Islands, Guinea Bissau. Trans R Soc Trop Med Hyg. 2021 Aug 2;115(8):847–53.

17. Waiswa C, Azuba R, Makeba J, Waiswa IC, Wangoola RM. Experiences of the one-health approach by the Uganda Trypanosomiasis Control Council and its secretariat in the control of zoonotic sleeping sickness in Uganda. Parasite Epidemiol Control. 2020 Nov;11:e00185.
